# Requirements for Human Cerebral Organoids

**DOI:** 10.1111/cpr.70201

**Published:** 2026-03-31

**Authors:** Ya‐Jie Xu, Chun‐Hui Duan, Tie‐Shan Tang, Yu‐Kai Wang, Weihong Song, Bo‐Qiang Fu, Ai‐Jin Ma, Bao‐Yang Hu, Zhen‐Ge Luo, Xiao‐Qing Zhang, Yan Liu, Zhao‐Qian Teng, Jie Hao, Qiang Wei, Zhi‐Hong Wu, Yong Zhang, Bing Zhao, Shan Bian, Jing‐Zhong Zhang, Bo Peng, Tian‐Qing Li, Yue‐Jun Chen, Jing Liu, Lei Wang, Yang Zhao, Wei‐Qi Zhang, Yao‐Jin Peng, Bo‐Ya Zhang, Xin‐Jie Bao, Yan‐Lin Wang, Yi Tang, Shi‐Jun Hu, Qian Li, Gao‐Xiang Zhu, Alex Zhang, Lijun Zhu, Qi‐Yuan Li, Tao Na, Lin Zhao, Yi‐Fang Ping, Hong‐Ling Zhao, Shuai‐Shuai Niu, Qi Zhou, Tong‐Biao Zhao, Chang‐Mei Liu

**Affiliations:** ^1^ Institute of Zoology, Chinese Academy of Sciences Beijing China; ^2^ Oujiang Laboratory, Center for Geriatric Medicine, International Center for Alzheimer’s Research, Prevention and Treatment, Key Laboratory of Alzheimer’s Disease of Zhejiang Province, The First Affiliated Hospital and Institute of Aging, Wenzhou Medical University Wenzhou China; ^3^ University of Chinese Academy of Sciences Beijing China; ^4^ Beijing Institute for Stem Cell and Regeneration Medicine Beijing China; ^5^ National Institute of Metrology Beijing China; ^6^ Standard Committee, Chinese Society for Cell Biology Shanghai China; ^7^ Beijing Technology and Business University Beijing China; ^8^ ShanghaiTech University Shanghai China; ^9^ Tongji University Shanghai China; ^10^ Nanjing Medical University Nanjing China; ^11^ Institute of Laboratory Animal Sciences, Chinese Academy of Medical Sciences and Comparative Medicine Center, Peking Union Medical College Beijing China; ^12^ Peking Union Medical College Hospital, Chinese Academy of Medical Sciences Beijing China; ^13^ Shenzhen HHLIFE Co., Ltd. Shenzhen China; ^14^ Nanchang University Nanchang China; ^15^ Suzhou Institute of Biomedical Engineering and Technology, Chinese Academy of Sciences Jiangsu China; ^16^ Fudan University Shanghai China; ^17^ Co‐Innovation Center of Neuro‐Regeneration Nantong University Nantong Jiangsu China; ^18^ Institute of Primate Translational Medicine, Kunming University of Science and Technology Kunming Yunnan China; ^19^ Center for Excellence in Brain Science and Intelligence Technology, Chinese Academy of Sciences Shanghai China; ^20^ Peking University Beijing China; ^21^ China National Center for Bioinformation and Beijing Institute of Genomics, Chinese Academy of Sciences Beijing China; ^22^ Organcare (Shenzhen) Biotechnology Company Shenzhen China; ^23^ Peking Union Medical College Hospital, Chinese Academy of Medical Sciences and Peking Union Medical College Beijing China; ^24^ The First Affiliated Hospital of Zhengzhou University Zhengzhou China; ^25^ Xuanwu Hospital, Capital Medical University, National Center for Neurological Disorders Beijing China; ^26^ Suzhou Medical College, Soochow University Suzhou China; ^27^ Qilu Pharmaceutical Co., Ltd Shandong China; ^28^ Shandong BioGenous Biological Technology Co., Ltd Shandong China; ^29^ Zephyrm Biotechnologies Co., Ltd. Beijing China; ^30^ Institute of Science and Technical Information of China Beijing China; ^31^ Shenzhen Medical Academy of Research and Translation Shenzhen Medical Academy of Research and Translation Shenzhen China; ^32^ National Institutes for Food and Drug Control Beijing China; ^33^ China National Institute of Standardization Beijing China; ^34^ Jinfeng Laboratory Chongqing China

“Requirements for Human Cerebral Organoids” is jointly drafted and agreed upon by experts from the Chinese Society for Stem Cell Research. This standard outlines the general requirements, technical requirements, testing methods, inspection rules, instructions for using, labeling, and transporting human cerebral organoids. It applies to the quality control for human cerebral organoids. This standard was originally released by the Chinese Society for Cell Biology on 28 October, 2024. We hope that the publication of these guidelines will promote institutional establishment, acceptance, and execution of proper protocols, and accelerate the international standardization of human cerebral organoid applications.

## 1 Scope

This document specifies the general requirements, technical requirements, testing methods, inspection rules, instructions for the use, labeling, and transportation of human cerebral organoids.

This standard is applicable for the in vitro preparation and inspection of cerebral organoids derived from human pluripotent or neural stem cells.

## 2 Normative References

The following content constitutes indispensable articles of this standard through normative reference. For dated references, only the edition cited applies. Only the latest edition (including all amendments) applies for undated references.

The “Pharmacopoeia of the People's Republic of China (2020 Edition, Volume III)”.

## 3 Terms and Definitions

The following terms and definitions apply to this document.

### 3.1 Organoids

In vitro three‐dimensional (3D) cellular cluster derived from stem cells, or progenitor cells, composed of organ/tissue‐specific cell types and capable of self‐renewal, self‐organization, and functionality akin to native tissues or organs [1, 2, 3, 4].

### 3.2 Human Cerebral Organoids

Organoids derived from human pluripotent stem cells or neural stem cells that contain a variety of cerebral cortical cell types including cortical neurons, intermediate progenitors, and radial glia [5, 6].

### 3.3 Neural Differentiation

The process by which pluripotent stem cells or neural stem cells progressively develop into various cell types, such as radial glial cells, intermediate progenitor cells, intermediate neurons, and cortical neurons, each characterized by unique morphological structures, gene expression profiles, and functional attributes [7].

### 3.4 Radial Glia Cell

Radial glial cells are a type of neural stem cells located in the human cortex, originating from neuroepithelial cells around the neural tube and capable of producing neurons and glial cells through both asymmetric and symmetric division during development [8, 9].

### 3.5 Intermediate Progenitor Cell

Intermediate progenitor cells, derived from radial glial cells essential for mature neuron production in the central nervous system, are characterized by the expression of EOMES, a versatile transcription factor crucial for regulating the proliferation and differentiation of neural stem cell [10].

### 3.6 Cortical Neuron

Cortical neurons, located in the cerebral cortex, are characterized by short axons and can be either excitatory or inhibitory, with some inhibitory interneurons being self‐generated and others migrating from ventral regions [11, 12].

## 4 Abbreviations

The following abbreviations apply to this document:

3D three dimension

Ct cycle‐threshold

DAPI 4,6‐diamino‐2‐phenylindole

PBS phosphate buffer saline

PCR polymerase chain reaction

RNA ribonucleic acid

STR short tandem repeat

## 5 General Requirements

### 5.1 Source Materials

The acquisition and use of source materials shall comply with domestically recognized ethical standards and local laws.

Research and production organizations focused on human cerebral organoids shall establish appropriate donor evaluation criteria based on the intended use.

### 5.2 Process and Information Management

Key factors affecting product quality during the acquisition, preparation, testing, transportation, and storage of human cerebral organoids shall be recorded. Unique identifiers shall be established to ensure traceability throughout the process.

The minimum retention time for records shall be specified to maintain the integrity and security of the record‐keeping system.

## 6 Technical Requirements

### 6.1 Morphology

Human cerebral organoids shall be spherical with visible edges under an optical microscope, and they shall display multiple layered “rosette” structures.

### 6.2 Chromosome Karyotype

The chromosome karyotype shall match the donor's karyotype.

### 6.3 Marker Genes

Human cerebral organoids shall express neural stem/progenitor cell marker genes like FOXG1/PAX6, FOXG1/SOX2, along with key genes associated with layer II‐VI cortical neurons in the cerebral cortex, such as REELIN, TBR1, CTIP2, CUX1, SATB2, BRN2, GLUT2, and TUJ1.

### 6.4 Cell Composition

The human cerebral organoids shall contain SOX2 and PAX6‐positive radial glial cells, EOMES‐positive intermediate precursor cells, and REELIN‐positive cortical neurons, along with neatly stratified II‐VI cortical neurons, including CUX1‐positive layer III‐II cortical neurons, TBR1 and CTIP2‐positive layer VI‐IV cortical neurons, SATB2‐positive layer IV‐II cortical neurons, BRN2‐positive layer II‐III and V cortical neurons, and TUJ1‐positive neurons.

After more than 72 days of differentiation, the TBR1‐positive cortical neurons shall make up no less than 20%. The CTIP2‐positive cortical neurons, as well as the BRN2‐positive cortical neurons, shall comprise no less than 20% and 3%, respectively. The SATB2‐positive cortical neurons shall also be present in no less than 3%.

### 6.5 Function Specifications

Cerebral organoids shall display the electrophysiological activity of mature neurons.

### 6.6 Cell Viability

When human cerebral organoids are differentiated for 60 days, the survival rate of cells in the organoids shall not be less than 70%.

### 6.7 Microorganisms

Testing of microorganisms, including fungi, bacteria, mycoplasma, and viruses shall be performed and documented.

### 6.8 STR

Shall align with the donor's STR.

## 7 Testing Methods

### 7.1 Morphology

The morphology of cerebral organoids cultured in vitro can be observed using an optical microscope.

### 7.2 Chromosome Karyotype

The karyotype can be determined according to the “Preparation and Quality Control of Animal Cell Matrix for the Production and Verification of Biological Products” in Part 3 of the 2020 edition of the Pharmacopoeia of the People's Republic of China.

### 7.3 Marker Genes

The marker gene expression can be detected using the method described in Supplementary Annex A.

### 7.4 Cell Composition

The cell compositions can be detected using the method described in Supplementary Annex B.

### 7.5 Function Specifications

The electrophysiological activity of cerebral organoids can be determined using either Patch‐clamp test or a microelectrode array assay as described in Supplementary Annex C and Supplementary Annex D respectively.

### 7.6 Cell Viability

The cell viability can be tested using the method described in Supplementary Annex B.

### 7.7 Microorganisms

#### 7.7.1 Fungi and Bacteria

According to the “1101 Sterility Test Method” in the Pharmacopoeia of the People's Republic of China (Part 3 of the 2020 edition).

#### 7.7.2 Mycoplasma

According to the “3301 Mycoplasma Test Method” in the Pharmacopoeia of the People's Republic of China (Part 3 of the 2020 edition).

#### 7.7.3 Exogenous Viral Factors

According to the “3302 Exogenous Viral Factor Test Method” in the Pharmacopoeia of the People's Republic of China (Part 3 of the 2020 edition).

### 7.8 STR

The method in Supplementary Annex E can be followed.

## 8 Instructions for Usage

The instructions for usage shall include, but not be limited to:

- Product name:
- Passage number;
- Cell number:
- Production date:
- Lot number:
- Production organization:
- Storage conditions:
- Shipping conditions:
- Contact information:
- Operation manual:
- Execution standard number;
- Manufacturing address;
- Postal code:
- Matters that need attention:

Note: According to user requirements, provide Endotoxin results.

## 9 Labels

The label shall include but not be limited to:

- Product name:
- Passage number;
- Cell number:
- Lot number:
- Production organization:
- Production date

## 10 Transportation

The appropriate mode of transportation and conditions should be selected to ensure the biological characteristics, safety, stability, and effectiveness of human cerebral organoids.

The transportation of human cerebral organoids should consider, but not be limited to, factors such as the characteristics of the organoids, the containers used, transport routes, transport duration, transport conditions, transport equipment, mode of transport, potential risks, and necessary safeguards.

The control measures for transportation conditions should include, but not be limited to temperature range, vibration control, contamination prevention, equipment performance, and suitable packaging.

Accompanying inspection and technical guidance documents should be provided upon user request.

The package shall be checked during transportation, and if necessary, additional freezing sources (e.g., dry ice and liquid nitrogen) should be added to maintain the appropriate transportation temperature.

## Author Contributions

Chang‐Mei Liu, Tong‐Biao Zhao, Qi Zhou, and Ya‐Jie Xu contributed to conception and design. Chang‐Mei Liu, Ya‐Jie Xu, Chun‐Hui Duan, Tie‐Shan Tang, Yu‐Kai Wang, and Qian Li drafted and revised the manuscript. Weihong Song, Bo‐Qiang Fu, Ai‐Jin Ma, Bao‐Yang Hu, Zhen‐Ge Luo, Xiao‐Qing Zhang, Yan Liu, Zhao‐Qian Teng, Jie Hao, Qiang Wei, Zhi‐Hong Wu, Yong Zhang, Bing Zhao, Shan Bian, Jing‐Zhong Zhang, Bo Peng, Tian‐Qing Li, Yue‐Jun Chen, Jing Liu, Lei Wang, Yang Zhao, Wei‐Qi Zhang, Yao‐Jin Peng, Bo‐Ya Zhang, Xin‐Jie Bao, Yan‐Ling Wang, Yi Tang, Shi‐Jun Hu, Gao‐Xiang Zhu, Alex Zhang, Lijun Zhu, Qi‐Yuan Li, Tao Na, Lin Zhao, Yi‐Fang Ping, Hong‐Ling Zhao, and Shuai‐Shuai Niu critically read and revised the manuscript.

## Supporting information


**Data S1:** cpr70201‐sup‐0001‐Supinfo.docx.

## Data Availability

The data that support the findings of this study are available from the corresponding author upon reasonable request.
