# Supplementary material for: Requirements for Human Cerebral Organoids
Source: Cell Prolif. 2026 Mar 31;59(5):e70201. doi: 10.1111/cpr.70201 (PMC13114783; doi:10.1111/cpr.70201)
Supplement: Supplementary file 1 — Data S1: cpr70201‐sup‐0001‐Supinfo.docx. [file CPR-59-e70201-s001.docx]

**Annex A**

**(Informative)**

**Real-time fluorescence quantitative PCR method for detection of cell type specific-marker genes**

**A.1 INSTRUMENTS**

A.1.1 Polymerase chain reaction (PCR) cycler.

A.1.2 Real-time fluorescence quantitative PCR cycler.

**A.2 REAGENTS**

Unless otherwise, all reagents used shall be analytical reagent, and the water used for testing shall be deionized water.

A.2.1 Phosphate buffered saline (PBS): pH 7.4.

A.2.2 Commercial RNA extraction kit.

A.2.3 Commercial RNA reversal transcription kits.

A.2.4 Commercial fluorescent quantitative PCR amplification kit.

A.2.5 qPCR primers for GAPDH and target genes.

**A.3 TESTING PROTOCOL**

A.3.1 Preparation of organoid samples

Aspirate the culture medium from organoids in vitro. Cerebral organoids were picked and washed for twice with PBS (A.2.1).

A.3.2 RNA extraction of organoids

Perform RNA extraction using the commercial RNA extraction kit (A.2.2) according to the manufacturer's instructions.

A.3.3 Organoid cDNA preparation

1 μg of RNA was obtained and perform cDNA synthesis the commercial RNA reverse transcription kit (A.2.3) according to the manufacturer's instructions.

A.3.4 Determination of gene expression

The organoid RNA reverse transcription product from step A.3.3 is used for gene expression determination. cDNA was amplified using the commercial fluorescence quantitative PCR amplification kit (A.2.4) and real-time fluorescence quantitative PCR instrument (A.1.2) and related qPCR primers (A.2.5) according to the manufacturer's instructions. Quantitative gene expression for GAPDH (CtG) and the target gene (CtM) were determined from the detection curve.

A.3.5 Analysis of target gene expression

Taking *GAPDH* expression as a reference, the expression level of the target genes shall be calculated as X = CtM/CtG.

**A.4 ANALYSIS OF RESULTS**

Repeat steps 4.3.1 to 4.3.5 for twice. Calculate the expression levels of target genes for three times, which are recorded as the average expression levels of organoid target genes.

**A.5 ACCURACY**

The absolute difference value of three independent tests shall not exceed 10% of their arithmetic mean under the same conditions.

**Annex B**

**(Informative)**

**Immunofluorescence Staining Method for Cell Composition Determination**

**B.1 INSTRUMENTS**

Laser confocal microscope.

**B.2 REAGENTS**

Unless otherwise, all reagents used shall be analytical reagent, and the water used for testing shall be deionized water.

B.2.1 Phosphate buffered saline (PBS): pH 7.4.

B.2.2 Commercial immunofluorescence staining kit.

B.2.3 Target proteins antibodies.

**B.3** **TESTING PROTOCOL**

B.3.1 Organoid sample preparation

Aspirate the culture medium from organoids in vitro. Cerebral organoids were picked and washed for twice with PBS (B.2.1).

B.3.2 Organoid immunofluorescence staining

Perform immunofluorescence staining of organoid with the commercial immunofluorescence staining kit (B.2.2) and the target antibody (B.2.3) following manufacturer's instructions using.

B.3.3 Imaging and analysis

Following instructions of microscope.

B.3.4 Target protein expression analysis

Target protein expression is calculated according to equation: X = N/M.

In the equation:

N-- number of stained cells

M-- total number of cells stained by DAPI

**B.4 ANALYSIS OF RESULTS**

The proportion of positive-staining cells shall be analyzed by averaging the proportion of target cell type from ≥ 30 organoids.

**Annex C**

**(Informative)**

**Patch-clamp test for electrophysiological activity of organoids (arbitration law)**

**C.1 INSTRUMENTS**

C.1.1 Vibrating microtome

C.1.2 Patch- clamp experimental system

**C.2 REAGENTS**

C.2.1 Low melting agarose

C.2.2 Cerebrospinal fluid

**C.3 SAMPLE PRESERVATION**

The organoids at day 120 - 200 during differentiation shall be embedded in 4% low melting agarose.

**C.4 TESTING PROTOCOL**

C.4.1 Sample slicing preparation

The ice-cold solution containing the following substances (in mM, pH adjusted to 7.4) was aerated with oxygen for 15 minutes: 92 NMDG, 1.3 NaH_2_PO_4_, 5 KCl, 0.5 CaCl_2_, 26 NaHCO_3_, 10 MgCl_2_, 5 sodium ascorbate, 2 thiosulfate, and 25 d-glucose. The embedded organoids were placed in the vibrating microtome, filled with the ice-cold solution. The thickness of the organoids was 200 µm, and the cutting speed was 0.1 mm/min. The organoid sections were maintained at room temperature for at least 1 hour to allow recovery from the mechanical shock induced by the slicing process.

C.4.2 mEPSC recording

For recording, the organoid sections were immersed in an external solution containing the following substances (in mM): 124 NaCl, 1.5 MgCl_2_, 3.3 KCl, 26 NaHCO_3_, 1.3 NaH_2_PO_4_, 11 glucose, and 2.5 CaCl_2_ at pH 7.4. This external solution was continuously equilibrated with 95% O_2_ and 5% CO_2_.

Recording microelectrodes (with resistance between 5 MΩ and 6 MΩ) were filled with intracellular solutions containing 115 mM cesium mesylate, 15 mM CsCl, 2 mM MgCl_2_, 10 mM HEPES, 10 mM EGTA, 4 mM Mg ATP, and 1 mM QX-314 at pH 7.4.

During mEPSC recording process, the cells were voltage-clamped at −60 mV. The organoid sections were recorded using MultiClamp 700B amplifier and Axon Digidata 1440A digitizer. To isolate AMPA receptor-mediated mEPSC, citroline (10 μM), tetrodotoxin (1 μM), and APV (2-amino-5-phosphonate, 50 μM) were added to the extracellular solution. All experiments were conducted at room temperature (23°C). Cell capacitance and series resistance were compensated. Data acquisition and analysis were performed using the pCLAMP Software Suite (version 10.6; Axon Instruments, CA, USA).

**C.5 ANALYSIS OF RESULTS**

All recorded neurons exhibited a transient inward current exceeding -30 mV after depolarization, which were blocked by tetrodotoxin. This inward voltage-gated Na^+^ current was followed by the activation of a more sustained K^+^ current. All recorded neurons reliably generated action potentials.

**Annex D**

**(Informative)**

**Detecting electrophysiological activity of organoids using microelectrode array (MEA)**

**D.1 INSTRUMENTS**

D.1.1 MEA porous culture plate

D.1.2 Maestro MEA equipment

**D.2 REAGENTS**

Matrigel matrix glue

**D.3 SAMPLE PRESERVATION**

The organoids at day 120 − 200 during differentiation were seeded into MEA porous culture plate.

**D.4 TESTING PROTOCOL**

D.4.1 Sample Slicing Preparation

The MEA culture plate was coated by Matrigel overnight. After removing the Matrigel, cerebral organoids shall be seeded into MEA culture plate and cultured for 5 − 20 minutes to ensure the adhesion. Medium (10−20 µL) was spread on the surface of the organoids. After incubating for 2 hours, at least 200 µL of medium was added.

D.4.2 Data recording

The optimal recording period of cerebral organoids patch was 7 to 14 days after culturing for 24 hours. The MEA culture plate was placed into the Maestro MEA system to record field potential (FP). Environmental conditions were maintained at 37°C and 5% CO_2_. Baseline data were recorded for 3 to 5 minutes after stabilizing for 10 minutes.

**D.5 ANALYSIS OF RESULTS**

The initial data was collected by AxIS Navigator and analyzed by Cardiac Analysis Tool and AxIS Metric Plotting Tool. The data indices shall be consistent with the results from patch-clamp experiments.

**Annex E**

**(Informative)**

**Organoid authentication by STR profiling**

**E.1 INSTRUMENTS**

E.1.1 Centrifuge

E.1.2 PCR Cycler

E.1.3 Electrophoresis apparatus

E.1.4 Micro ultraviolet spectrophotometer

**E.2 REAGENTS**

E.2.1 Cell DNA extraction kit

E.2.2 STR cell identification kit

**E.3 SAMPLE PRESERVATION**

The sample shall be stored at -80°C after processing.

**E.4 TESTING PROTOCOL**

E.4.1 Sample preparation

Cerebral organoids were picked and subjected to centrifugation to remove the supernatant.

E.4.2 Extraction of DNA

a) Perform DNA extraction from organoids according to the instructions of the Cell DNA extraction kit.

b) DNA shall yield an A260/A280 ratio of 1.8 − 2.0 measured by micro ultraviolet spectrophotometer.

c) DNA volume ≥20 μL, DNA concentration ≥50 ng/μL.

E.4.3 PCR amplification

a) Performed amplification of STR loci according to standard PCR amplification methods or the commercially approved kit instructions.

b) A negative control, sample detection group, and a positive control shall be added. Sterile water shall be the template for PCR amplification in the negative control group; use the DNA extracted from organoid and primary tumor tissue samples as templates in the sample detection group; use a DNA template with a confirmed STR profiling as the positive control group.

c) Detect the PCR products of three groups by agarose gel electrophoresis. Clear target band shall be observed in positive control but not in the negative control group.

E.4.4 STR genotyping

Detect PCR products by capillary electrophoresis gene analyzer and STR genetic map data shall be obtained. The PCR banding pattern of organoids and primary tumor tissue shall be consistent.

**E.5 ANALYSIS OF RESULTS**

E.5.1 If the STR locus contain the same number of repeats, only one allele peak shall appear in the profile. If the STR locus contain different number of repeats, two allele peaks shall appear in the profile. The test is considered effective when no allelic peaks appeared in the negative control group, and the positive control group is consistent with its standard genotyping data.

E.5.2 If more than two allelic peaks appear at the STR locus of the tested sample, the sample shall be determined to be cross-contaminated after repeated tests to exclude interfering factors such as mutations in the primer binding region, provided that the test is valid.
